# Silicon Nanocrystals with pH-Sensitive Tunable Light Emission from Violet to Blue-Green

**DOI:** 10.3390/s17102396

**Published:** 2017-10-20

**Authors:** Jing Wang, Junhong Guo, Jing Chen

**Affiliations:** 1 Department of Electronic Science and Engineering, Nanjing University of Posts and Telecommunications, Nanjing 210003, China; jhguo@njupt.edu.cn (J.G.); jchen@njupt.edu.cn (J.C.); 2 National Laboratory of Solid State Microstructures and Department of Physics, Nanjing University, Nanjing 210093, China; 3 Jiangsu Provincial Engineering Laboratory for RF Integration and Micropackaging, Xinmofan Street 66, Nanjing 210003, China

**Keywords:** silicon, nanocrystals, pH sensitive, photoluminescence

## Abstract

We fabricated a silicon nanocrystal (NC) suspension with visible, continuous, tunable light emission with pH sensitivity from violet to blue-green. Transmission electron microscopy (TEM) images and X-ray diffraction (XRD) pattern analysis exhibit the highly crystalline nanoparticles of silicon. Photoluminescence (PL) spectra and photoluminescence excitation (PLE) spectra at different pH values, such as 1, 3, 5, 7, 9, and 11, reveal the origins of light emission from the silicon NC suspension, which includes both the quantum confinement effect and surface bonding. The quantum confinement effect dominates the PL origins of silicon NCs, especially determining the tunability and the emission range of PL, while the surface bonding regulates the maximum peak center, full width at half maximum (FWHM), and offsets of PL peaks in response to the changing pH value. The peak fitting of PLE curves reveals one of the divided PLE peaks shifts towards a shorter wavelength when the pH value increases, which implies correspondence with the surface bonding between silicon NCs and hydrogen atoms or hydroxyl groups. The consequent detailed analysis of the PL spectra indicates that the surface bonding results in the transforming of the PL curves towards longer wavelengths with the increasing pH values, which is defined as the pH sensitivity of PL. These results suggest that the present silicon NCs with pH-sensitive tunable light emission could find promising potential applications as optical sources, bio-sensors, etc.

## 1. Introduction

The research on low-dimensional silicon nanostructures is an active research field, due to the interesting chemical and physical properties [1,2,3,4,5]. Of all the low-dimensional silicon nanomaterials, the silicon NCs with a variety of types, such as porous silicon [6,7,8], silicon/SiO_2_, or silicon/silicon nitride nuclear shell nanostructure [9,10,11], soluble quantum dots [12,13,14,15,16,17], silicon NC-based photonic crystal slabs [18], silicon NC ultrathin films [19], etc., have drawn increasing attention. In particular, the silicon NCs with intense light emission take up an important place in a large number of applications, including light-emitting sources [18,20,21], sensors [22], detectors [13,23,24], bio-tagging [22,25], bio-imaging [21,26], photovoltaics [27], optical communication [28], spintronics devices [29], etc.

Based on varied approaches of fabricating silicon NCs, including chemical vapor deposition [30], the non-thermal plasmas approach [20,31,32], electrochemical etching [13,32,33], chemical dissolution [16,34], annealing [35,36,37], pulsed laser ablation [15,38,39], and scanning transmission electron microscopic lithography [29], etc., various colors of silicon NC luminescence have been obtained, such as infrared [11,37], red [17,38], orange [13,40], yellow [16], green [13,41], blue [14,15,16,23,42,43,44], or multiple colors [21,45], etc. However, it is still a challenge to achieve controllable blue light emission of free silicon NCs without clustering, since the sizes of silicon NCs have to be much smaller than excitation Bohr radius of 4.3 nm [28] based on the quantum confinement effect [15,30,46,47]. On the other hand, the light emission from extensive origins suppress the luminescence originating from the quantum confinement effect as a result of the ultra-active surfaces of silicon NCs [48], although the sizes of NCs are small enough to show the quantum confinement effect [6,49]. Due to the high surface/volume ratio and the size confinement effects, the surface of silicon NCs are so active that they are often bonded to atoms or groups, such as hydrogen [16,30,34,35,41,42,50], oxygen [9,20,33,36,43,47], hydroxyl [46], or others [38]. Therefore, the blue luminescence that is often obtained is attributed to defects [44], surface states [48], interface localized states [36,43,51], oxidation [43], etc. There derives a question that, for silicon NCs with ultra-small sizes, such as 2 nm, how do the bondings of NCs affect the light emission features? Free silicon NCs are often dispersed in different types of solvent, many of which have plenty of groups [5,52,53,54,55], such as methyl groups [32,54], 1-dodecene [54], etc., especially hydrogen atoms [54,56] or hydroxyl groups [56,57]. Since hydrogen atoms and hydroxyl groups are basic components of water and almost everywhere in cells, studying how the pH value influences the light emission of silicon NCs becomes significant. If the light emission characters of silicon NCs can be changed by simply adjusting the pH value, the silicon NCs will be widely applicable in bio-sensors [58], imaging [5], etc.

In this article, we report a pH-sensitive photoluminescence of silicon NCs, which covers from violet to blue-green. The bonds between surface silicon atoms of NCs and hydrogen atoms/hydroxyl groups make the PL transform towards longer wavelengths, corresponding to the increasing pH value, and the PL spectra are stronger in mild (neutral, weakly acidic, or alkalescent) environments than others, closer to the PL spectra based on the quantum confinement effect in silicon NCs. PLE curves group into two parts when the environment is alkaline. The cause of the pH sensitive photoluminescence is explored by the analysis of PLE peak fitting. It is inferred that there is a special state responsible for the surface bonding which causes the offsets of PL and PLE at different pH values, although the origins of light emission are dominated by the quantum confinement effect. Considering the mild intracellular environment, together with the biochemical processes in cells happening mostly in a narrow pH range, the present silicon NCs with the pH-sensitive luminescence features from pH values 3 to 11 could find potential applications in a broad range, such as probes [23,54,58], monitors [17], tags [5,17,21], etc.

## 2. Materials and Methods

**Figure 1:**
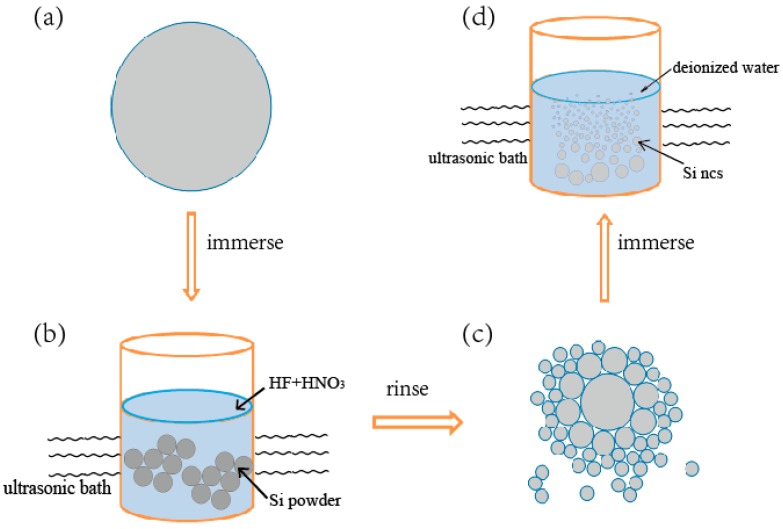
Schematic of the silicon NC fabrication process: (**a**) A grain of silicon powder; (**b**) silicon powder immersed in an ultrasonic bath with aqueous solution; (**c**) a silicon grain becomes loose after corroding; and (**d**) loose silicon grains are broken into suspended silicon NCs after another ultrasonic treatment with water.

Details of the synthesis process of the silicon NC suspension is illustrated in Figure 1. In brief, we first immerse 4.0 g silicon powder (99.9%, 10 μm, Weiye Chemical Company, Ltd., Yancheng, China) in an ultrasonic bath for 30 min, with 40 mL aqueous solution of 1 mL hydrofluoric acid (AR, 40.0% by volume, Macklin Company, Ltd., London, UK) and 0.02 mL nitric acid (AR, 65% by volume, Macklin Company, Ltd., London, UK). Thus, we obtained loose grains by corroding the silicon powder. After rinsing the corroded silicon powder with deionized water 4–5 times until the pH value of the supernatant liquid reaches 4, we added 10 mL deionized water into the corroded silicon powder and continued the ultrasonic treatment for 60 min. Finally the liquid as left to stand for at least 30 min, and we obtained the supernatant fluid with silicon NCs suspended in it as our silicon NCs colloid sample. In the experiment, we can adjust the pH value of the silicon NC colloids by adding an aqueous solution of HCl (18% in mass diluted from the 36% HCL(AR), produced by Xuhong Chemical Company, Ltd. Changzhou, China) and NaOH (20% in mass, from the 98% NaOH(AR) pieces produced by Macklin Company, Ltd., London, UK).

Surface morphologies of silicon NCs were characterized by a JEOL JEM-2100 TEM after a drop of silicon suspension was dried on a piece of copper mesh. A quantity of silicon NC suspension was dried in a nitrogen atmosphere as the XRD sample. The XRD used in the present work is an ARL X’TRA diffractometer. The PL and PLE spectra were obtained by an FLS 920 fluorescence spectrometer (Edinburgh Instruments Company, Ltd., Edinburgh, UK), using a 450 W xenon lamp as the excitation optic source.

## 3. Results and Discussion

### 3.1. Morphology Characterization and Size Statistics

**Figure 2:**
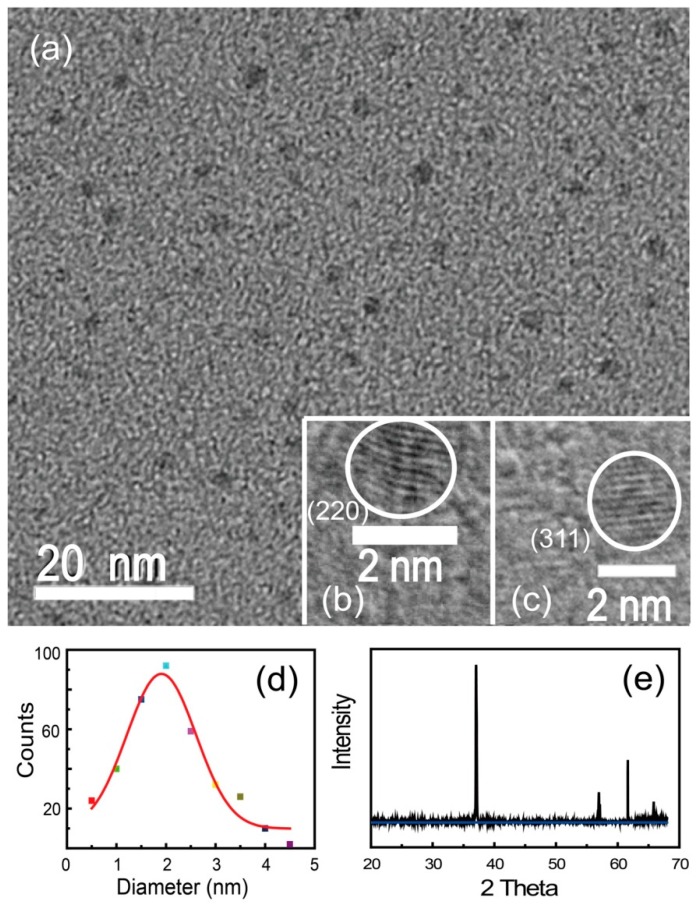
(**a**) A TEM image of silicon NCs; (**b**,**c**) HRTEM of silicon NCs, with lattice fringes corresponding to (220) and (311) planes of silicon; (**d**) size statistic of silicon NCs from a large number of TEM images. The most probable size of1.91 nm is calculated according to Gaussian fitting, as the curve shows; and (**e**) XRD line of silicon NCs.

The TEM images of silicon NCs are taken at the accelerating voltage of 200 kV, as shown in Figure 2a. It is clearly seen that the silicon NCs appear close to spheres and disperse everywhere without gathering. We count the numbers of silicon NCs with different diameters from a number of TEM images. Most silicon NCs are between 1 nm and 4 nm in diameter, and no silicon NC larger than 4.5 nm is observed. The counting statistic with a Gauss fitting is shown in Figure 2d, suggesting that the most probable diameter is about 1.91 nm. Since the silicon NCs smaller than 1 nm are easy to miss, the actual most probable diameter is probably much less than 1.91 nm.

Figure 2b,c present the high-resolution TEM (HRTEM) images of silicon NCs, which display two highly-crystalline silicon NCs, with lattice fringes corresponding to (220) and (311) planes of silicon. As can be seen from Figure 2e, the sharp XRD line indicates the high crystallization and good monodispersion of silicon NCs, consistent with the HRTEM images, exhibiting typical peaks of silicon nanostructures [12,44,59,60].

### 3.2. PL and PLE Spectral Characteristic

**Figure 3:**
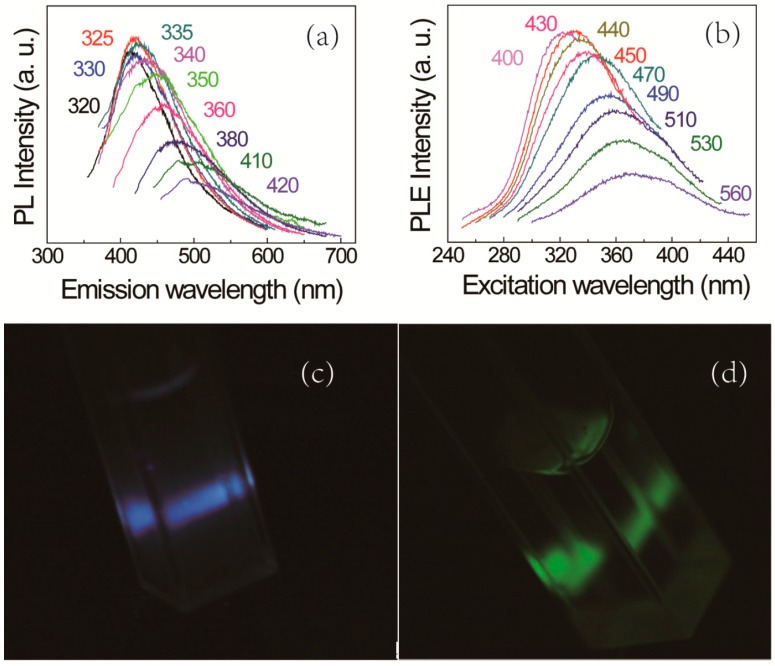
(**a**) PL spectra of silicon NCs colloid with pH value of 4; (**b**) PLE spectra of silicon NCs colloid with pH value of 4; (**c**) optical photograph obtained from silicon NC colloids excited at 360 nm, corresponding to blue (~450 nm); and (**d**) optical photograph from silicon NCs colloid excited at 420 nm, corresponding to green color (~510 nm).

We study the PL spectra and PLE spectra acquired from silicon NC colloids with different pH values in detail. As shown in Figure 3a, the PL spectra from silicon NC colloids with an unadjusted pH value, which is 4, indicate the strongest PL peak responding to the excitation wavelength of 325 nm. There is a redshift in the light emission with the increasing excitation wavelength. When the excitation wavelength varies from 320 nm to 420 nm, the PL peak wavelength increases from 400 nm to 520 nm. When silicon NCs with probable size can always be excited by the wavelengths smaller than 325 nm, the intensity of PL peak increases. As the number of silicon NCs which can be excited decreases along with continuing increase of excitation wavelength, the PL intensity reduces and a small water Raman peak appears. According to Wolkin's model [6] the most probable diameter can be estimated from the most intensive PL peak wavelength, which is around 1.8 nm, agreeing with the size statistic shown in Figure 2d. Despite the bulk silicon band gap is 1.12 eV (1107 nm), the PL signal is too weak to be observed beyond the emission wavelength 510 nm, implying the absence of silicon NCs larger than 4 nm in diameter, which is also consistent with Figure 2d. Since most of silicon NCs are much smaller than the excitation Bohr radius of bulk silicon around 4.3 nm [22], we can conclude that the redshift along with increasing excitation wavelength is attributed to the quantum confinement effect in silicon NCs. The redshift of PLE peak center accompanied by increasing emission wavelength, as shown in Figure 3b, further confirm the result that the quantum confinement effect results in the tunable light emission of silicon NCs. Figure 3c,d show the optic photos of lighting silicon NC colloids at the exciting wavelength of 360 nm and 420 nm, respectively. It is evident that the light emission of the resulted silicon NCs colloid is strong enough to be observed by the naked eye.

**Figure 4:**
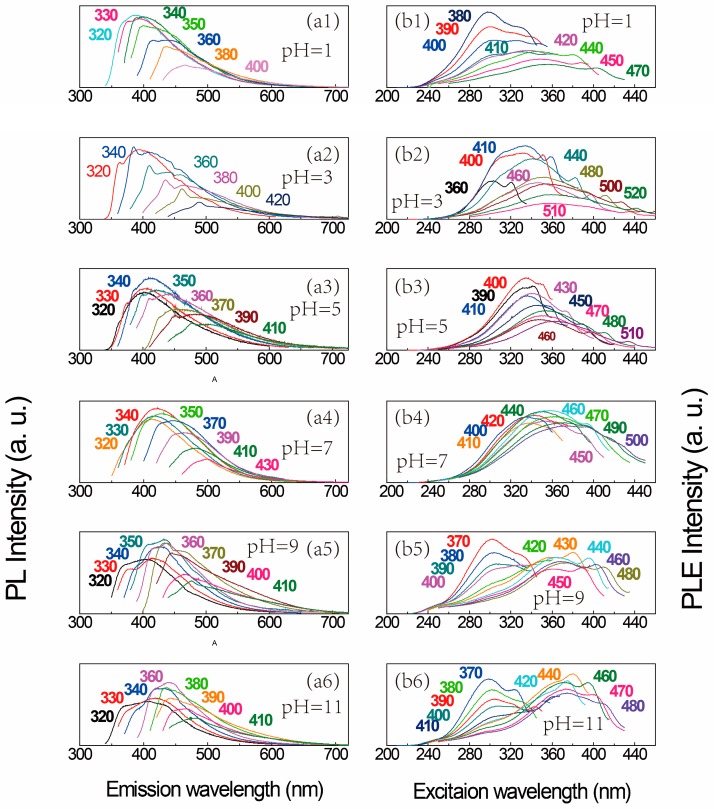
PL spectra from silicon NCs colloid with pH values of (**a1**) 1, (**a2**) 3, (**a3**) 5, (**a4**) 7, (**a5**) 9, and (**a6**) 11. PLE spectra from silicon NCs colloid with pH values of (**b1**) 1, (**b2**) 3, (**b3**) 5, (**b4**) 7, (**b5**) 9, and (**b6**) 11.

The redshift of the PL peak with increasing excitation wavelength can also be observed from silicon NC colloids with pH 1, 3, 5, 7, 9 and 11, as shown in Figure 4(a1–a6). After pH 11, the PL spectrum is difficult to analyze due to the chaotic and weak curves, which is not exhibited in the figure. As can be seen, the light emission is still visible, and the emission wavelength gradually redshifts from 400 nm to 500 nm with the excitation wavelength increasing from 320 nm to 430 nm. If we take the PL spectrum shown in Figure 3a as a basic spectrum, the offsets of PL peaks corresponding to the same excitation wavelength in Figure 4a1–a6 vary from approximately 5 nm to 25 nm. As a result, the PL shapes in Figure 3 and Figure 4 are similar despite on the various pH values of silicon NC colloids, meaning that the quantum confinement effect plays the most important role in the light emission of silicon NCs. However, besides the offsets of PL peaks, the PL peak center reaches the maximum intensity at 320, 340, 340, 340, 350, and 360 nm, corresponding to the pH 1, 3, 5, 7, 9, and 11, respectively, which is difficult to be interpreted as the quantum confinement effect. With increasing pH values, not only the maximum PL peak, but also nearly every PL curve at the same excitation wavelength, transforms toward longer wavelengths, as displayed in Table 1 (based on Figure 4(a1–a6)), and the PL FWHM at the same excitation wavelength becomes wider. The PL peaks shift toward longer wavelengths, which we define as the pH sensitivity of photoluminescence, showing that the light emission of silicon NCs could be influenced by the chemical environment.

PLE spectra of silicon NCs colloid with different pH values are presented in Figure 3b and Figure 4(b1–b6). In Figure 3b, the PLE peak wavelength increase monotonically with the excitation wavelength, as well as Figure 4(b3,b4) indicate, implying that the same wavelength can excite silicon NCs with different sizes. However, the shift of the PLE peak cannot be easily observed when the pH value is 1 or 3, as shown in Figure 4(b1,b2), because there is a raised shoulder in the PLE curve around the excitation wavelength of 300 nm. Considering the acidic environment, it is inferred that the shoulder is related to the surface bonding between silicon NCs and hydrogen atoms. Similar to Figure 3b, a small redshift of the PLE peak with the increasing emission wavelength can be observed in the long wavelength area (which is from 420 nm to 470 nm in Figure 4(b1) and is from 440 nm to 500 nm in Figure 4(b2)), suggesting that the light emission feature could be influenced by an acidic environment only in the high energy part. The PLE spectrum in Figure 4(b5,b6) displays more significant differences from the others. The PLE curves separate into two groups. PLE curves in group 1 are assembled near the ultraviolet area, approximately covering from 250 nm to 350 nm in excitation wavelength, in response to an emission wavelength from 370 nm to 410 nm. No obvious shift is observed, but the PLE peak is gradually weakened with the rising emission wavelength. Compared to group 1, the PLE peak suddenly rise at the emission wavelength of 420 nm, meaning two origins of silicon NC’s light emission. PLE curves in this group (group 2) include a few curves located in the violet-blue area, with PLE peaks from 350 nm to 400 nm, corresponding to emission wavelengths from 420 nm to 480 nm. A small redshift of the PLE peak can be observed in pace with increasing emission wavelength, accompanied by decreasing intensity and width. The shape and location of the PLE curves in group 2 are similar to the ones in Figure 3b, matching the PL spectrum in Figure 4(a5,a6), respectively, much better than group 1. It seems that an alkaline environment influences the PLE performance more in the short wavelength region, consistent with the PL spectra.

**Figure 5:**
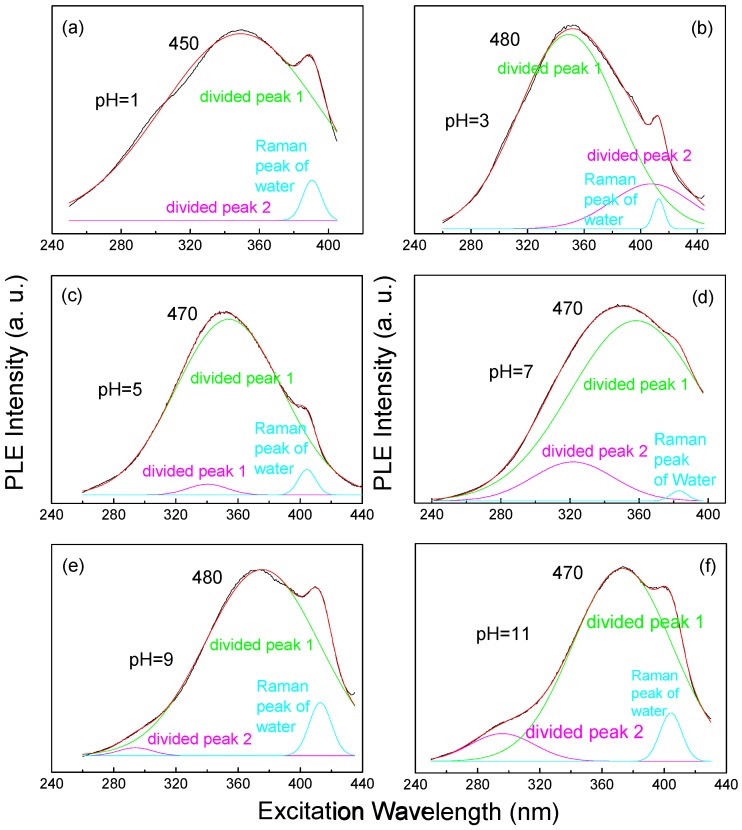
Gauss fitting of PLE spectra with the silicon NC colloid pH values at (**a**) 1, at the emission wavelength 450 nm; (**b**) 3, at the emission wavelength 480 nm; (**c**) 5, at the emission wavelength 470 nm; (**d**) 7, at the emission wavelength 470 nm; (**e**) 9, at the emission wavelength 480 nm; and (**f**) 11, at the emission wavelength 470 nm.

The PLE spectra with the features above suggest two origins of silicon NC photoluminescence. At different pH values, it will be found that a typical PLE curve (picking from Figure 4(b1–b6)) such as that shown in Figure 5a–f could be divided into two peaks by peak fitting besides the Raman peak of water. A typical PLE curve should be representative and strong enough for peak fitting. Therefore, for a pH value 1, the curve of a 450 nm emission wavelength is chosen and for pH values of 3, 5, 7, 9, and 11, the curves at the emission wavelengths of 470 nm or 480 nm are chosen. The center wavelengths of two divided peaks at different pH values are listed in Table 2. It is observed that, at the same emission wavelength, one of the peaks (divided peak 1) redshifts with the pH value increasing from 1 to 11 and the other peak (divided peak 2) has a shortening center wavelength with the pH value increasing (the divided peak 2 does not appear at pH 1). The divided peak 2 is not visible when the pH is 1, as shown in Figure 5a, maybe because the divided peak 2 should be longer than 400 nm, beyond the range of the PLE. The shift of divided peak 1 with an increasing pH value is far less intense than the shift of the divided peak 2 and the divided peak 1. Considering the center wavelength of the divided peak 1, as well, it is reasonable to conclude that divided peak 1 is due to the quantum confinement effect [46], while the divided peak 2 is inferred to be based on the surface bonding between silicon NCs and hydrogen atom or hydroxyl group. The divided peak 2 appears more intense in the short emission wavelength range (shorter than 410 nm, roughly), especially in an alkaline environment. As a result, when the pH value is 9 or 11, responding to the same emission wavelengths, the peak wavelengths of the group 1 PLE curves are shorter compared to the PLE peak wavelengths at pH 1–7, while the peak wavelengths of the group 2 PLE curves are longer. Therefore, it is explained that the grouping of PLE curves at the pH values 9 or 11 is a result of the divided peak 2 redshift to the short wavelength area. Although distinct grouping cannot be observed in the PLE curves at pH 1–7, we can still find clues suggesting the two origins of the silicon NC photoluminescence, which is consistent with the PLE spectra of pH 9 and 11. As Figure 5b1–b4 shows, when the pH value is 1, 3, 5, or 7, the divided peak 2 is in a longer wavelength area, which cannot show the obvious grouping of PLE curves. When the emission wavelength is the same, the PLE peak wavelength at pH 1 is shorter than it is at pH 3 and the PLE peak wavelength at pH 3 is shorter than it is at pH 5, etc., meaning the divided peak 1 is dominant in an acid environment and divided peak 2 gradually shows its power as the pH value increases, and is even weak in a neutral environment. Thus, we can conclude that the PLE patterns at different pH values are the combined effect of the divided peaks 1 and 2. In another word, the PL of silicon NCs originates both from the quantum confinement effect and the surface bonding between NCs and hydrogen atoms or hydroxyl groups. Another result of this combined effect is that, in the low energy area, the PL and PLE curves show more intense changes compared to a neutral environment.

The comparison among PLE spectra at different pH values implies that once the acid environment disappears, the divided peak 2 shifts to a shorter wavelength area, which is more effective in the higher energy part, resulting in a strong grouping of PLE curves in an alkaline environment. Based on the analysis of the PLE spectra above, the PL transforming towards longer wavelengths is easy to understand. Based on the statistics in Table 2, the shift of the divided peak 1 is not obviously corresponding to the same emission wavelength when the pH value changes. Thus, the change of the PLE curve patterns responsible for the pH value increasing depends mainly on the divided peak 2. The divided peak 2 shifts towards the shorter wavelength with the increasing pH value, meaning the corresponding excitation wavelength is longer at the same emission wavelength. That is, when the pH value increases, the emission wavelength shifts towards longer wavelengths at the same excitation wavelength. In another word, the PL peak will shift toward a longer wavelength in pace with the ascending pH value.

All of the above features of the PL and PLE spectra indicate that both strong acidic and alkalescent environment can greatly change the characteristics of silicon NC colloid photoluminescence, which means some electron states are formed based on the dangling bonds between hydrogen atoms or hydroxyl groups and silicon atoms on the NCs’ surfaces, although the quantum confinement effect dominates the light emission of silicon NCs, consistent with Figure 3a and Figure 4(a4). The high energy part is influenced greatly by the chemical environment, indicating that smaller silicon NCs with more active surfaces could produce dangling bonds more easily. Smaller NCs have rough surfaces, which makes them bond more to hydroxyl groups. This is why the divided peak 2 from the PLE curve is more effective in the higher energy part. It consequently implies that the content of hydrogen atom and hydroxyl group bonding to NCs’ surfaces influence the light emission properties. It is found that when the content of hydrogen atoms and hydroxyl groups achieve a rough balance, the spectra appears as a typical effect of quantum confinement [46], and the light emission properties reach an optimum with a wider emission range and higher intensity, comparatively. Based on the near-neutral environment in biological cells, the silicon NCs could be applied to tracing biological processes, keeping their intrinsic characteristics. Since weak acidity-alkalinity switching often happens in bio-cells, the pH sensitivity of silicon NCs’ luminescence in the routine pH range from 1 to 11 of the cell environment [60,61] is probably helpful for monitoring biological processes.

## 4. Conclusions

We have demonstrated that, by monodispersed ultra-small silicon NCs with active surfaces, the tunable photoluminescence from violet to blue-green with pH sensitivity is observed due to both the quantum confinement effect and the bonding interaction between silicon atoms on the NCs’ surfaces and hydrogen atoms or hydroxyl groups. It is discovered that there is a special surface state based on the bonding between silicon NCs and hydroxyl groups, which creates a large effect in an alkaline environment, widening the PL and PLE curves, driving PL curves to transform towards longer wavelengths with the increasing pH value. In a word, the quantum confinement effect makes the silicon NCs show tunable violet-blue-green photoluminescence, while the surface state makes the photoluminescence sensitive to the pH value. The exploration of the pH sensitivity of silicon NCs’ luminescence could help further research about the effects of surface bonding on the energy states, such as pH sensitivity of other silicon nanostructures, or more complicated groups bonding to silicon NCs, even groups bonding to other silicon-based nanomaterials, etc. The tunable photoluminescence make silicon NCs possible to work as optical sources [26], nano-optoelectronic devices [62,63], nano-optoelectronic devices, and intracellular tracing [62]. Additionally, the pH sensitivity of luminescence from silicon NCs makes it possible to realize real-time monitoring on biological processes in cells [62,64]. Since hydrogen and hydroxyl appear nearly everywhere in biology cells and participate in most biochemical processes, the silicon NCs bonding with hydrogen or hydroxyl show excellent bio-compatibility that they are easy to enter bio-cells without rejection [5,24,62], providing a beautiful prospect in bio-probes and bio-sensors, even bio-light emitters. Furthermore, considering the multiple PL colors from the silicon NCs, it is also possible to monitor the pH value, which can greatly help us understand detailed processes in chemical or biochemical reactions.

## Figures and Tables

**Table 1:** The center wavelengths of PL peaks at different pH values corresponding to various excitation wavelengths.

PL Peak Wavelength	pH = 1	pH = 3	pH = 5	pH = 7	pH = 9	pH = 11
320	381	390	400	405	402	410
340	400	407	412	420	424	429
350	-	-	414	425	-	-
360	430	433	436	-	443	447
370	-	-	450	450	450	-
380	455	460	-	-	-	-
390	-	-	460	464	462	464

**Table 2:** The center wavelengths of two divided peaks corresponding to typical PLE curves at different pH values.

pH Value	Emission Wavelength (nm)	Center Wavelength of Divided Peak 1	Center Wavelength of Divided Peak 2
1	450	349	N/A
3	480	355	405
5	470	358	343
7	470	361	322
9	480	375	297
11	470	373	293

**Figure A1:**
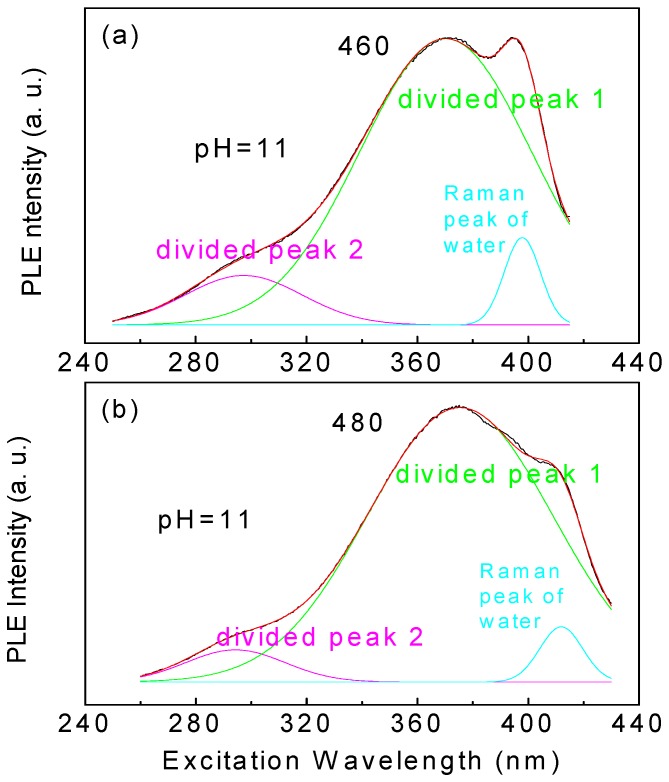
Gauss fitting of the PLE spectra at the emission wavelength of (**a**) 460 and (**b**) 480 nm when the pH value of silicon NCs colloid is 11.

## Appendix A

Figure A1 is an example to show the redshift of divided peak 1 in response to the increasing excitation wavelength. The centered wavelength of divided peak 1 at the excitation wavelength 460 nm is 370 nm, as Figure A1a shows, while the centered wavelength at the excitation wavelength 480 nm is 377 nm, as Figure A1b shows.
